# Associations between ADHD and risk of six psychiatric disorders: a Mendelian randomization study

**DOI:** 10.1186/s12888-024-05548-y

**Published:** 2024-02-05

**Authors:** Yanwei Guo, Junyao Li, Renqin Hu, Huirong Luo, Zheng Zhang, Jinglan Tan, Qinghua Luo

**Affiliations:** Department of Psychiatry, the First Affiliated Hospital of Chongqing Medical University, Chongqing, China

**Keywords:** Attention deficit hyperactivity disorder, Autism spectrum disorder, Schizophrenia, Mendelian randomization study

## Abstract

**Background:**

Observational studies and diagnostic criteria have indicated that Attention Deficit Hyperactivity Disorder (ADHD) frequently comorbid with various psychiatric disorders. Therefore, we conducted a Mendelian randomization (MR) study to explore this potential genetic association between ADHD and six psychiatric disorders.

**Methods:**

Using a two-sample Mendelian randomization (MR) design, this study systematically screened genetic instrumental variables (IVs) based on the genome-wide association studies (GWAS) of ADHD and six psychiatric disorders, with the inverse variance weighted (IVW) method as the primary approach.

**Results:**

The study revealed a positive and causal association between ADHD and the risk of ASD, with an odds ratio (OR) of 2.328 (95%CI: 1.241–4.368) in the IVW MR analysis. Additionally, ADHD showed a positive causal effect on an increased risk of schizophrenia, with an OR of 1.867 (95%CI: 1.260–2.767) in the IVW MR analysis. However, no causal effect of Tic disorder, Mental retardation, Mood disorders and Anxiety disorder with ADHD was found in the analysis mentioned above.

**Conclusion:**

Our MR analysis provides robust evidence of the causal role of ADHD in increasing the risk of ASD and schizophrenia. However, ADHD is not associated with the risk of Tic Disorder, Mental Retardation, Mood Disorders and Anxiety Disorder. This suggests the need for increased attention to the co-occurrence of ADHD-ASD or ADHD-schizophrenia and the implementation of timely intervention and treatment.

**Supplementary Information:**

The online version contains supplementary material available at 10.1186/s12888-024-05548-y.

## Introduction

Attention Deficit Hyperactivity Disorder (ADHD) is a childhood-onset neurodevelopmental disorder with a prevalence of about 1.4%-3.0%, exhibiting significant gender differences. This condition is characterized by inappropriate development, impairing inattention, motor hyperactivity, and impulsivity [1]. There are phenotypic similarities between ADHD and other neurodevelopmental syndromes, all of which are associated with cognitive impairment. They are more common in males and often associated with varying degrees of developmental delay, neurological soft signs, and motor abnormalities [2]. Additionally, ADHD is often associated with comorbidities with other psychiatric disorders, including neurodevelopmental disorders such as Autism Spectrum Disorder (ASD), Mental Retardation, Tic Disorder, as well as other psychiatric disorders such as Schizophrenia, Mood Disorders, and Anxiety Disorder [3, 4].

Comorbidity within mental disorders is pervasive and generally occurs at a higher rate than expected by coincidence, with the risk persisting over time [5]. The presence of comorbidities may lead to more adverse outcomes, hindering the individual's social performance and influencing the prognosis. Additionally, it often masks the pre-existing disease, resulting in more complex clinical manifestations and affecting the treatment plan and efficacy [6]. Data from clinical studies indicate that in their lifetime, 65 to 89 percent of adults with ADHD may have one or more additional psychiatric disorders, including mood and anxiety disorders. Similarly, 60 to 100 percent of children with ADHD are likely to have comorbid psychiatric disorders such as anxiety, mood disorders, tic disorders, and developmental disorders, consistent with rates observed in adults [7]. The specific underlying mechanisms are not fully understood, but based on the available evidence, ADHD-intrinsic vulnerability and genetic factors may play role.

Recent research on the root causes of mental illness has revealed that many of the same genes contribute to seemingly different diseases, and they are intricately interconnected and mutually influential, with no clear dividing line [8]. The genomic variation of ADHD was found to be nonspecific, and the aggregated polygenic risk scores of a series of common variants showed that the genomic variation of ADHD significantly overlapped with diseases such as ASD, Schizophrenia, and affective disorders [9]. In other words, "If you have ADHD, you may be much more likely to have another psychiatric disorder." The study of Mendelian randomization may be an effective way to explore this problem.

Mendelian randomization aims to study the causal relationship between two factors using genetic variation as an instrumental variable [10]. Generally, single nucleotide polymorphisms (SNPs) are the most used genetic variants. Since SNPs are formed during embryonic development and are independent of the environment and other acquired factors, the MR design provides a randomization procedure that also minimizes the interference of residual confounding and reverse causation [11]. In recent years, with further exploration of MR research methods, it has become increasingly confirmed as an ideal way to study and the pathogenic association between two complex diseases [12].

The purpose of this study was to explore the potential causal associations between ADHD and six psychiatric disorders (ASD, Mental Retardation, Tic Disorder, Schizophrenia, Mood Disorders, and Anxiety Disorder) based on MR analyses in a European population. Simultaneously, our study will provide a foundation for developing prevention strategies for the other six psychiatric disorders following individuals with ADHD in practice.

## Methods and materials

**Fig. 1:**
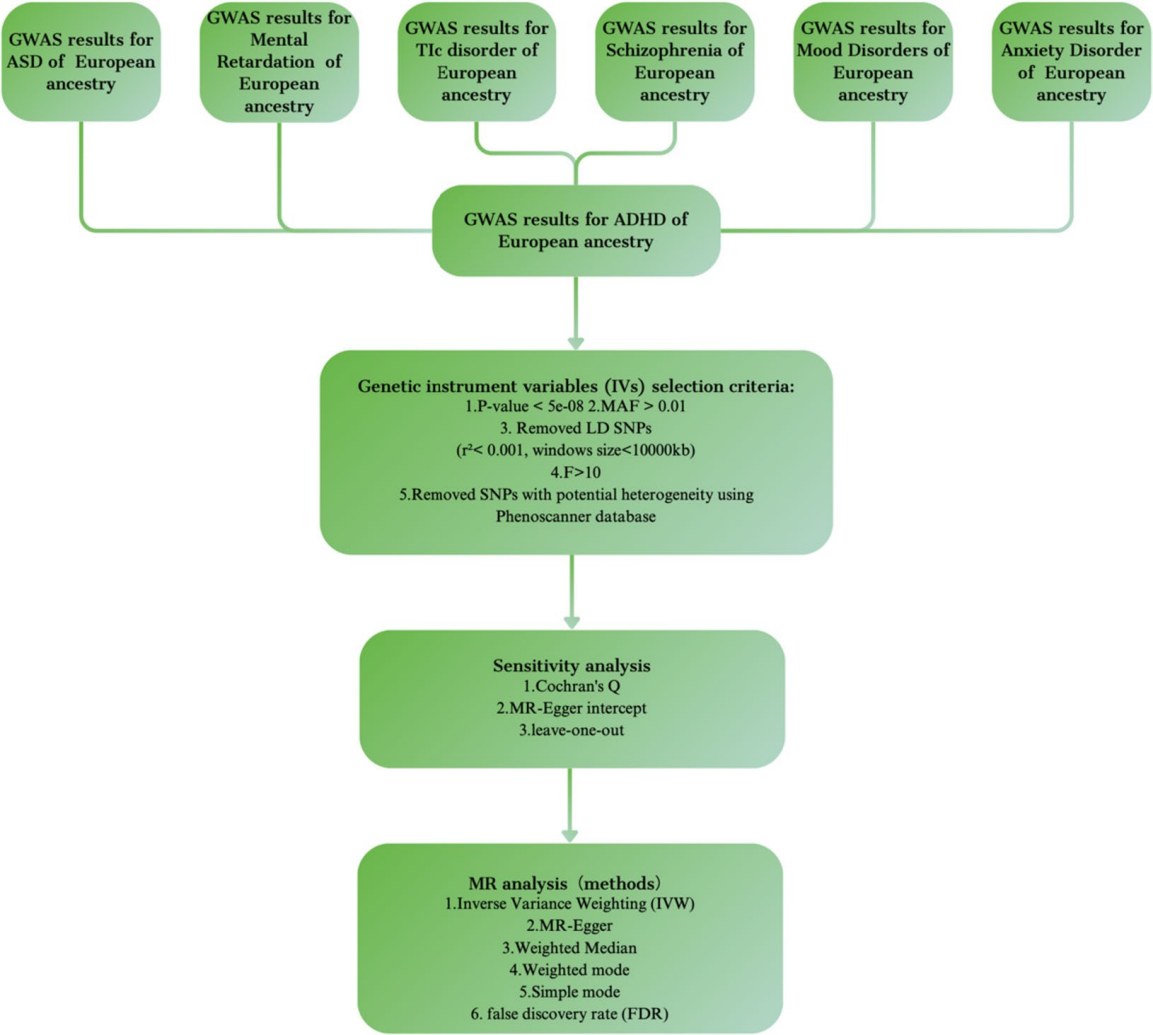
Study design and overview of our Mendelian randomization (MR) study. ADHD Attention Deficit Hyperactivity Disorder, ASD Autism Spectrum Disorder, IVW inverse-variance weighted

The study design overview of the MR is presented in Fig. 1. In this study, we used inverse variance weighted (IVW) as the primary method of analysis to estimate the causal effects of ADHD and other psychiatric disorders. It's worth noting that all datasets involved in this research were publicly available, and ethical approvals were obtained for all original papers. You can find more detailed information in Table 1.

**Table 1:** Characteristics of participants and power calculation for each association between ADHD and six psychiatric disorders risk

Trait	Data sources	Year	Ancestry	F
**Outcome:**	**IEU GWAS ID**		European	
ASD	ieu-a-1185 finn-b-F5_MENTRET finn-b-F5_TIC ieu-b-5102 finn-b-KRA_PSY_MOOD finn-b-KRA_PSY_ANXIETY	2017		37.14
Mental Retardation		2019		36.91
Tic Disorder		2021		36.91
Schizophrenia		2022		34.53
Mood Disorders		2021		36.91
Anxiety Disorder		2021		36.91
**Exposure**	**EBI database**			
ADHD	GCST90275136	2023	European	

*ASD* Autism Spectrum Disorder, *ADHD* Attention Deficit Hyperactivity Disorder, *EBI The* European Bioinformatics Institute

### Source of data

To ensure the high efficacy of MR analysis in estimating causal effects and the reproducibility of results, we utilized publicly available genome-wide association study (GWAS) data for the present research. Ethical approvals were obtained for all original papers, and more detailed information can be found in Table 1.

### ADHD

We utilized GWAS summary data on ADHD exposure from the EBI database, with the ADHD-related SNPs obtained from a comprehensive GWAS meta-analysis of the iPSYCH cohort, comprising 21,738 ADHD cases and 36,548 controls of European ancestry [13]. The study proposed the age-dependent liability threshold (ADuLT) model-based GWAS and compared it with SPACox and standard case–control GWAS in simulations under two generative models and with varying degrees of ascertainment, as well as in the iPSYCH cohort. Each analysis was restricted to the individuals in the subcohort and the cases for the specific phenotype being analyzed. Ultimately, the study found that ADuLT identifies independent genome-wide significant associations in ADHD [14].

### Six psychiatric disorders

Through a search of the MRC-IEU database, we identified SNPs associated with various psychiatric disorders, including ASD, Tic Disorder, Mental Retardation, Schizophrenia, Mood Disorders, and Anxiety Disorder, with data available until October 2023.

### Selection of instrumental variants (IVs)

The genetic instrumental variables for exposure were obtained from the EBI database and encompassed a total of 58,286 participants, adhering to the following screening criteria for relevant SNPs: (1) a significance level of p < 10^–8^ indicating a high degree of SNP and ADHD correlation, (2) Linkage disequilibrium (LD) describing the correlation between genetic variants, typically caused by the physical proximity of genetic variants to each other [11]. The identified SNPs were clumped for LD using PLINK with a stringent cutoff of clumping R2 = 0.001 within a window of 10,000 kb. (3) The F-statistic was calculated to assess the strength of the IVs. Following calculation, all F-statistics were found to be greater than 10, thereby avoiding the occurrence of weak IV bias. Data were extracted from the two databases and then combined with the exposed effect values and corresponding results for the same effect alleles. Information was collected for each SNP, including the major allele, allele frequency, beta coefficient, P-value, and standard error (SE). Subsequently, we harmonized the exposure and outcome datasets, removing palindromic and weak instrumental variants, and utilized the remaining SNPs to conduct MR analysis. Additionally, we utilized PhenoScanner V2, a comprehensive database of human genotype–phenotype associations from publicly available genetic association studies [15], to assess whether the selected SNPs were associated with confounders (P < 5 × 10–5) in the relationship between ADHD and the six psychiatric disorders. If confounding factors were identified, would be adjusted in further analysis.

### Statistical analysis

The TwoSampleMR package (version 0.5.7) in R (version 4.3.1) was utilized for statistical analysis. Prior to conducting the TwoSampleMR analysis, the Instrumental Variable (IV) needed to satisfy three key assumptions: (1) it must be closely associated with the exposure factors, (2) it should not be related to potential confounding factors, and (3) it must not be influenced by factors other than the exposure. We employed Inverse Variance Weighting (IVW), MR-Egger, Weighted Median, and Weighted Model for the TwoSampleMR analysis, with statistical significance indicated by a P-value of less than 0.05. IVW emerged as the predominant method due to its industry-standard utilization in pooling data for MR analyses, obviating the need for individual-level data to calculate causal effect sizes by directly employing pooled data [11]. The IVW method provides reliable estimates when all IVs are valid. It includes both fixed-effects IVW and random-effects IVW. In cases where heterogeneity exists in the MR analysis, we will apply the random-effects IVW, which is less susceptible to bias from weaker SNP-exposure associations [16]. Additionally, scatter plots, forest plots, and funnel plots were generated to visually represent results.

The methodology employed in this study utilizes weighted regression, where the weight for fitting is determined as the reciprocal of the outcome variance. Additionally, the intercept term is excluded from the regression analysis. In the context of gene pleiotropy identification, the MR-Egger regression's intercept term is scrutinized, and evidence of horizontal pleiotropy is indicated by a significance level of *P* < 0.05.

To assess heterogeneity, the Cochran's Q statistic is used, with a significance level of *P* < 0.05 to identify its presence of heterogeneity. Furthermore, visual representations of the results are generated using scatter plots, forest plots, and funnel plots. A sensitivity analysis is conducted using leave-one-out plots, where each SNP is sequentially eliminated to assess the impact of the remaining SNPs on the association between the exposure and outcome. The objective is to evaluate the potential influence of specific SN on the outcome.

In our study, we investigated multiple exposures, and the P-values obtained from IVW were adjusted using the false discovery rate (FDR) correction method. We considered differences to be statistically significant when the adjusted P-value was less than 0.05. High-confidence findings were those that survived multiple-testing adjustment. We computed the odds ratios (ORs) and their corresponding 95% confidence intervals (CIs) for the risk of six psychiatric disorders associated with a one-unit change in exposure.

Anonymized study data for the primary analyses presented in this report are available upon request from any qualified investigator for the purpose of replicating the results.

## Results

**Fig. 2:**
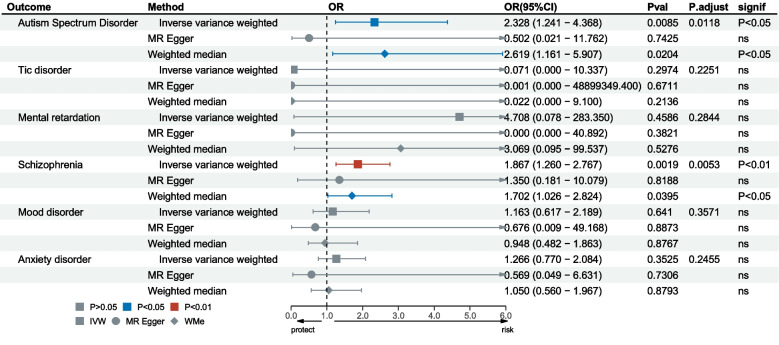
The causal relationship between ADHD and six psychiatric disorders. OR odds ratio. CI confidence interval

Figure 2 presents a forest plot that visually displays the results of three MR methods for ADHD associated with six psychiatric disorders. We recommend that IVW results be considered the primary method for analyzing causal associations between exposure factors and outcome variables. This study demonstrated a positive and causal association between ADHD and the risk of ASD, with an OR of 2.328 (95%CI: 1.241–4.368) in the IVW MR analysis. The weighted median method also yielded consistent and similar estimates that supported the risk effect of ADHD on ASD, with an OR of 2.619 (95%CI: 1.161–5.907). However, no statistical significance was found for the MR Egger approach. None of the three analyses revealed any causal effect of Tic disorder, Mental retardation, Mood disorders, or Anxiety disorder on BD. Furthermore, ADHD had a positive causal effect on an increased risk of Schizophrenia, with an OR of 1.867 (95%CI: 1.260–2.767) in the IVW MR analysis. The weighted median method estimated a consistent OR of 1.702 (95%CI: 1.026–2.824) that supported the risk effect of ADHD on Schizophrenia, while the MR Egger approach did not find statistical significance.

**Fig. 3:**
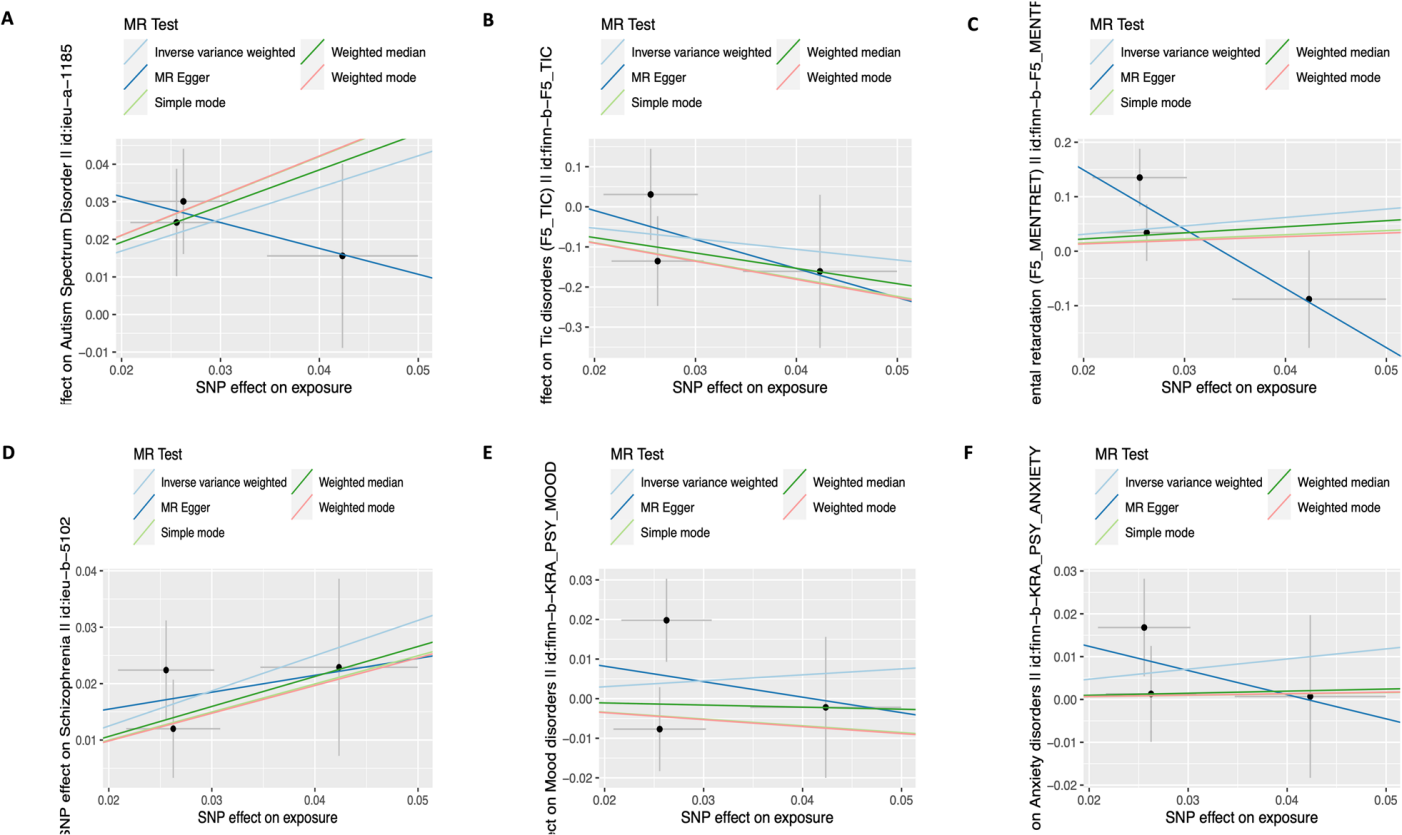
Scatter plot of SNPs associated with ADHD and risk of six psychiatric disorders. **A** Autism Spectrum Disorder (ASD). **B** Tic disorder. **C** Mental Retardation. **D** Schizophrenia. **E** Mood Disorders. **F** Anxiety Disorder

Figure 3 displays scatter plots of the estimated causal effect of each SNP on six psychiatric disorders, using five MR methods. The plot illustrates the SNP effects on ADHD and six psychiatric disorders, with the y-axis representing the log odds ratio (OR) and 95% confidence intervals. The regression slopes of the lines represent the causal estimates using the five approaches (inverse-variance weighted (IVW), MR-Egger, weighted median, simple mode, and weighted mode) in Mendelian randomization (MR) analysis.

**Fig. 4:**
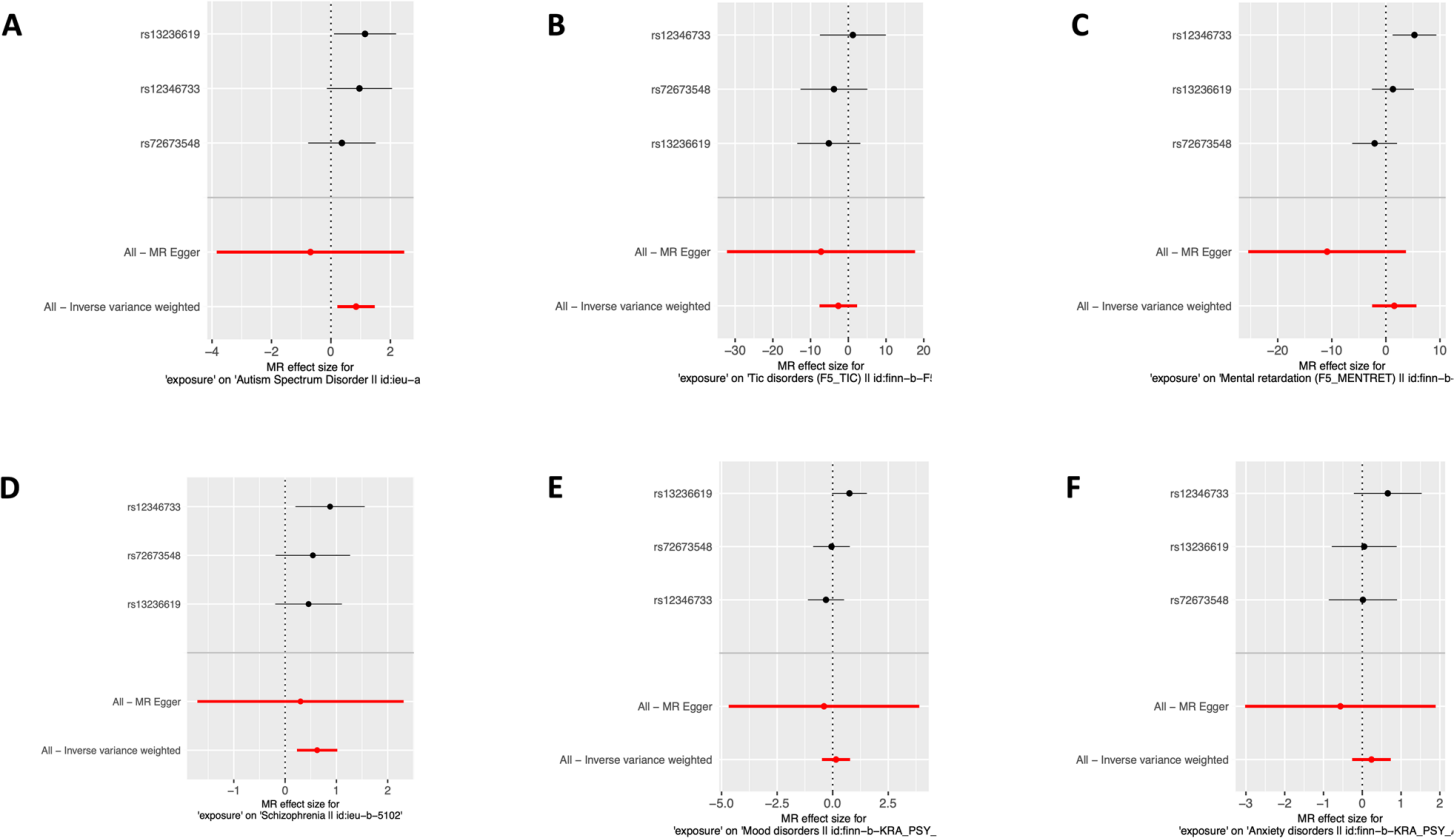
Forest plot of causal associations between ADHD and risk of six psychiatric disorders. **A** Autism Spectrum Disorder (ASD). **B** Tic disorder. **C** Mental Retardation. **D** Schizophrenia. **E** Mood Disorders. **F** Anxiety Disorder

In the forest plot of Fig. 4, each horizontal solid line reflects the results of individual SNP estimation using the Wald ratio method. The combined results indicate that individuals with ADHD were more likely to be at risk for ASD or Schizophrenia.

### Sensitivity analysis

Both the Cochran’s MR-Egger regression analysis and IVW analysis Q indicated no heterogeneity in SNPs between ADHD and ASD (MR-Egger *p* = 0.761, IVW *p* = 0.595), between ADHD and Tic disorder (MR-Egger *p* = 0.312, IVW *p* = 0.560), between ADHD and Schizophrenia (MR-Egger *p* = 0.391, IVW *p* = 0.657), between ADHD and Mood Disorders (MR-Egger *p* = 0.062, IVW *p* = 0.157), and between ADHD and Anxiety Disorder (MR-Egger *p* = 0.344, IVW *p* = 0.516). However, heterogeneity was observed between ADHD and Mental Retardation (MR-Egger *p* = 0.206, IVW *p* = 0.044). According to the MR-Egger intercept, we identified and corrected for horizontal pleiotropy, and no evidence of pleiotropy was found. To further validate the results, a leave-one-out analysis was conducted to assess whether the causal effect of exposures on outcomes was biased by any single SNP [17]. The results of the leave-one-out analysis of ADHD and ASD, Tic disorder, Schizophrenia, and Anxiety Disorder showed that the results of the IVW analysis after the sequential exclusion of individual SNPs were consistent with the results of the IVW analysis that included all SNPs, indicating that no single SNP had a strong effect on results (S1).

## Discussion

This study is the first to analyze the association between ADHD and multiple psychiatric disorders using MR and has found evidence of a causal association between ADHD and ASD and schizophrenia. The present study suggests that individuals with ADHD may have an increased risk of developing ASD and schizophrenia. However, no causal relationship was found between ADHD and Mental Retardation, Tic Disorder, Mood Disorders, as well as Anxiety Disorder. Additionally, sensitivity analysis found no significant interference with heterogeneity and horizontal pleiotropy [18].

ADHD and ASD are both highly inherited neurodevelopmental disorders. There is evidence that the two disorders frequently co-occur and share cognitive abnormalities related to temporal foresight [19]. Observational studies have indicated that 20–50% of children with ADHD meet the criteria for ASD, while 30–80% of children with ASD meet the criteria for ADHD [20]. Patients with a dual diagnosis of ADHD-ASD often exhibit more severe symptoms than those with either diagnosis alone. In clinical diagnosis and treatment, it can be challenging to fully distinguish between the two conditions. In the previous Diagnostic and Statistical Manual of Mental Disorders (DSM)-III-R and DSM-IV, ADHD and ASD could not be diagnosed simultaneously. However, in the DSM-V, the possibility of a co-diagnosis of ADHD and ASD was clarified [21].

Our article offers a new diagnostic and treatment approach for ADHD, ASD, or SCZ. When doctors diagnose patients with ADHD, they may consider the possibility of comorbid ASD or SCZ. This approach could potentially change the doctors' diagnostic and treatment, leading to improved patient outcomes. In terms of pharmacological treatment, some patients with comorbid ASD may have difficulty tolerating ADHD medications, but they can be very helpful in overall disease management. The use of central nervous system stimulants can start with small doses and gradually increase to achieve therapeutic effects. If intolerable, alternative ADHD medications can be. For patients with comorbid SCZ, In the case of confirmed comorbidity, prioritize treating SCZ to understand its impact on cognitive and functional impairments. Establish a baseline to evaluate the potential effects of ADHD treatment on symptoms. If significant inattention persists post-psychiatric treatment, explore non-stimulant options like atomoxetine or guanfacine, and consider non-pharmacological interventions such as psychoeducation cognitive-behavioral therapy [22].

The mechanism of comorbidity between ADHD and ASD is not fully understood. Firstly, observational findings have indicated that offspring of mothers with ADHD have a 2.5-fold increased risk of developing ASD compared to those of mothers without ADHD. Similarly, siblings with ASD have a 3.7-fold increased risk of developing ADHD [23, 24]. Additionally, the results of several twin studies on ASD and ADHD traits provide strong evidence that genetic factors play a role in the clinically established co-occurrence of ASD and ADHD [25–27]. Based on an observational study that found a significant impact of the environment on ADHD-ASD comorbidity, it was noted that patients living with their mothers had a higher likelihood of comorbidities [24, 28]. The association between ASD and ADHD was found to be weaker in paternal half-siblings than in maternal half-siblings, suggesting that environmental factors shared by relatives contribute to the co-occurrence of these diseases. This is because maternal and paternal half-siblings share the same number of co-isolated genes, but maternal half-siblings share a greater degree of family environment. This explanation is based on the observation that, after their parents separate, most children tend to live primarily with their mothers [29]. Neuroimaging studies have also compared prefrontal function in four groups of youth: those with ASD, ADHD, comorbid ASD and ADHD, or neither disorder (controls) using a temporal discounting task and fMRI. The non-control group exhibited both common anomalies and unique features. Specifically, the comorbid group showed unique and more severe impairments affecting the lateral and ventromedial prefrontal cortex, ventral striatum, and anterior cingulate cortex, compared to the non-comorbid and control groups [19]. These pathophysiological findings shed light on the fact that ASD-ADHD comorbidity is not simply a combination or addition of both disorders; it is neurofunctionally distinct. Further research is needed to obtain more accurate valid results.

There are limited studies on the comorbidity of ADHD and schizophrenia. At the molecular level, dopamine is considered to be the primary neurotransmitter involved in the pathophysiology of both ADHD and schizophrenia [30]. There is strong evidence indicating that individuals with ADHD have reduced dopaminergic metabolic activity, and decreased dopamine function is particularly associated with impulsivity. Conversely, there is increasing evidence that hyper dopamine in individuals with schizophrenia is specifically linked to positive psychotic symptoms of the disorder [31]. Observational studies have found that patients with schizophrenia often exhibit clinically relevant symptoms of inattention and hyperactivity, and ADHD is relatively common among children with a genetic risk for schizophrenia [32, 33]. In imaging studies, differences in the dynamics of the cortex in schizophrenia and ADHD may be related to differences in the functional connectivity of specific sensory vs. association thalamic nuclei [34]. In the realm of social psychology, due to ADHD, children are often perceived as troubled adolescents in school, experience poor classroom discipline and academic performance, and are excluded by their peers. However, parents typically only notice their children's overt behaviors and may employ improper educational methods, such as physical punishment or neglecting to provide proper guidance, which can exacerbate the child's hyperactivity, social withdrawal, passive resistance, and even dissociation, potentially leading to the appearance of symptoms resembling schizophrenia [30, 35].

There are several highlights in our MR study. Firstly, to the best of our knowledge, this is the first study to evaluate the genetic association between ADHD and six other psychiatric disorders based on a two-sample MR analysis with large-scale GWAS data. Compared to previous observational studies, the major merit of the MR design is to minimize biases caused by confounding and reverse causality, thus enhancing causal inference [36]. Secondly, we performed causal estimates in two large databases to ensure consistency and thus obtained credible causal inferences. The population of our study was limited to individuals of European ancestry, which minimized bias due to population stratification. Furthermore, we systematically screened confounding factors associated with ADHD and the other six psychiatric disorders using the PhenoScanner database and eliminated IVs associated with these confounding factors to avoid potential horizontal pleiotropy of genetic IVs. Additionally, Cochran’s Q test and the leave-one-out method were employed to examine heterogeneity in the IVs. If the Cochran’s Q test detected no significant heterogeneity, we utilized the IVW linear regression for unbiased association estimation. Finally, in addition to employing the IVW method as the primary analysis approach, various MR complementary methods were used to ensure result accuracy, including the MR Egger, weighted median, weighted mode, and mode methods.

However, we would like to acknowledge some limitations. Firstly, this study utilized publicly available data from the EBI database and the FinnGen databases to control the bias caused by sample overlap, but there is still potential overlap due to the large sample size and the restriction to the European region [37]. Secondly, although a series of strict steps were taken to identify outlier variants to avoid horizontal pleiotropy, we were still unable to completely eliminate the impact of horizontal pleiotropy, which may be attributed to the complex and unclear biological function of many genetic variants. Lastly, while GWAS could provide new insights into genes involved in ADHD and six psychiatric disorders, further studies are needed to better understand the pathophysiology. Mendelian randomization (MR) methods provide an initial assessment of the causal relationships, but they do not fully explain underlying biological mechanisms.

## Conclusion

Overall, our MR analysis provides strong evidence of the causal role of ADHD in increasing the risk of ASD and schizophrenia, while not showing an association between ADHD and the risk of Tic Disorder, Mental Retardation, Mood Disorders and Anxiety Disorder. This suggests that we should pay more attention to the co-occurrence of ADHD with ASD or schizophrenia and implement intervention and treatment as early possible. At the same time, genetic screening for susceptibility genes related to ADHD may be a future direction.

### Supplementary Information

**Additional file 1.**

## Data Availability

All data used in this study are publicly available. To assess the data, please contact the corresponding author.
