# Supplementary material for: Associations between ADHD and risk of six psychiatric disorders: a Mendelian randomization study
Source: BMC Psychiatry. 2024 Feb 5;24:99. doi: 10.1186/s12888-024-05548-y (PMC10840247; doi:10.1186/s12888-024-05548-y)
Supplement: Supplementary file 1 — Additional file 1. [file 12888_2024_5548_MOESM1_ESM.docx]

Confounders

We excluded according to the characteristics reported. Once one uncorrelated trait was found would be excluded. The SNP shown in the table below may represent more than one trait.

| outcome | snp | confunder |
| --- | --- | --- |
| ASD | rs11210887 | Educational attainment |
|  |  | Age at menarche |
|  |  | Age completed full time education |
|  |  | Current tobacco smoking |
|  |  | Fluid intelligence score |
|  |  | Qualifications: A levels or as levels or equivalent |
|  |  | Qualifications: college or university degree |
|  |  | Qualifications: none |
|  |  | Smoking status: current |
|  |  | Schizophrenia |
|  |  | Years of educational attainment |
|  | rs12705966 | Sleep duration |
|  |  | Types of physical activity in last 4 weeks: light diy |
|  |  | Wheeze or whistling in the chest in last year |
|  | rs4261436 | Intelligence multi trait analysis |
|  |  | Arm fat mass left |
|  |  | Arm fat mass right |
|  |  | Arm fat percentage left |
|  |  | Arm fat percentage right |
|  |  | Body fat percentage |
|  |  | Body mass index |
|  |  | Fluid intelligence score |
|  |  | Leg fat mass left |
|  |  | Leg fat mass right |
|  |  | Trunk fat mass |
|  |  | Whole body fat mass |
|  | rs4813421 | Arm fat-free mass left |
|  |  | Arm fat-free mass right |
|  |  | Arm predicted mass left |
|  |  | Arm predicted mass right |
|  |  | Basal metabolic rate |
|  |  | Trunk fat-free mass |
|  |  | Trunk predicted mass |
|  |  | Whole body fat-free mass |
|  |  | Whole body water mass |
|  | rs4916723 | Alcohol intake frequency |
|  | rs57806515 | Arm fat-free mass left |
|  |  | Arm predicted mass left |
|  |  | Basal metabolic rate |
|  |  | Comparative body size at age 10 |
|  |  | Impedance of arm left |
|  |  | Impedance of arm right |
|  |  | Impedance of leg left |
|  |  | Impedance of leg right |
|  |  | Impedance of whole body |
|  |  | Trunk fat-free mass |
|  |  | Trunk predicted mass |
|  |  | Whole body fat-free mass |
|  |  | Whole body water mass |
|  | rs704061 | Arm fat mass left |
|  |  | Arm fat mass right |
|  |  | Arm fat percentage left |
|  |  | Arm fat percentage right |
|  |  | Body fat percentage |
|  |  | Body mass index |
|  |  | Hip circumference |
|  |  | Leg fat mass left |
|  |  | Leg fat mass right |
|  |  | Leg fat percentage left |
|  |  | Leg fat percentage right |
|  |  | Leg fat-free mass left |
|  |  | Leg fat-free mass right |
|  |  | Leg predicted mass left |
|  |  | Number of operations |
|  |  | Overall health rating |
|  |  | Qualifications: college or university degree |
|  |  | Trunk fat mass |
|  |  | Trunk fat percentage |
|  |  | Waist circumference |
|  |  | Weight |
|  |  | Whole body fat mass |
|  | rs7563362 | Body mass index males |
|  |  | Body mass index |
|  |  | Age at menarche |
|  |  | Arm fat mass left |
|  |  | Arm fat mass right |
|  |  | Arm fat percentage left |
|  |  | Arm fat percentage right |
|  |  | Arm fat-free mass left |
|  |  | Arm fat-free mass right |
|  |  | Arm predicted mass left |
|  |  | Arm predicted mass right |
|  |  | Basal metabolic rate |
|  |  | Body fat percentage |
|  |  | Body mass index |
|  |  | Comparative body size at age 10 |
|  |  | Comparative height size at age 10 |
|  |  | Hip circumference |
|  |  | Impedance of arm left |
|  |  | Impedance of arm right |
|  |  | Impedance of leg left |
|  |  | Impedance of leg right |
|  |  | Impedance of whole body |
|  |  | Leg fat mass left |
|  |  | Leg fat mass right |
|  |  | Leg fat percentage left |
|  |  | Leg fat percentage right |
|  |  | Leg fat-free mass left |
|  |  | Leg fat-free mass right |
|  |  | Leg predicted mass left |
|  |  | Leg predicted mass right |
|  |  | Trunk fat mass |
|  |  | Trunk fat percentage |
|  |  | Trunk fat-free mass |
|  |  | Trunk predicted mass |
|  |  | Waist circumference |
|  |  | Weight |
|  |  | Whole body fat mass |
|  |  | Whole body fat-free mass |
|  |  | Whole body water mass |

| outcome | snp | confunder |
| --- | --- | --- |
| Mental Retardation | rs11210887 | Educational attainment |
|  |  | Age at menarche |
|  |  | Age completed full time education |
|  |  | Current tobacco smoking |
|  |  | Fluid intelligence score |
|  |  | Qualifications: A levels or as levels or equivalent |
|  |  | Qualifications: college or university degree |
|  |  | Qualifications: none |
|  |  | Smoking status: current |
|  |  | Schizophrenia |
|  |  | Years of educational attainment |
|  | rs12705966 | Sleep duration |
|  |  | Types of physical activity in last 4 weeks: light diy |
|  |  | Wheeze or whistling in the chest in last year |
|  | rs4261436 | Intelligence multi trait analysis |
|  |  | Arm fat mass left |
|  |  | Arm fat mass right |
|  |  | Arm fat percentage left |
|  |  | Arm fat percentage right |
|  |  | Body fat percentage |
|  |  | Body mass index |
|  |  | Fluid intelligence score |
|  |  | Leg fat mass left |
|  |  | Leg fat mass right |
|  |  | Trunk fat mass |
|  |  | Whole body fat mass |
|  | rs4813421 | Arm fat-free mass left |
|  |  | Arm fat-free mass right |
|  |  | Arm predicted mass left |
|  |  | Arm predicted mass right |
|  |  | Basal metabolic rate |
|  |  | Trunk fat-free mass |
|  |  | Trunk predicted mass |
|  |  | Whole body fat-free mass |
|  |  | Whole body water mass |
|  | rs4916723 | Alcohol intake frequency |
|  | rs57806515 | Arm fat-free mass left |
|  |  | Arm predicted mass left |
|  |  | Basal metabolic rate |
|  |  | Comparative body size at age 10 |
|  |  | Impedance of arm left |
|  |  | Impedance of arm right |
|  |  | Impedance of leg left |
|  |  | Impedance of leg right |
|  |  | Impedance of whole body |
|  |  | Trunk fat-free mass |
|  |  | Trunk predicted mass |
|  |  | Whole body fat-free mass |
|  |  | Whole body water mass |
|  | rs704061 | Arm fat mass left |
|  |  | Arm fat mass right |
|  |  | Arm fat percentage left |
|  |  | Arm fat percentage right |
|  |  | Body fat percentage |
|  |  | Body mass index |
|  |  | Hip circumference |
|  |  | Leg fat mass left |
|  |  | Leg fat mass right |
|  |  | Leg fat percentage left |
|  |  | Leg fat percentage right |
|  |  | Leg fat-free mass left |
|  |  | Leg fat-free mass right |
|  |  | Leg predicted mass left |
|  |  | Number of operations |
|  |  | Overall health rating |
|  |  | Qualifications: college or university degree |
|  |  | Trunk fat mass |
|  |  | Trunk fat percentage |
|  |  | Waist circumference |
|  |  | Weight |
|  |  | Whole body fat mass |
|  | rs7563362 | Body mass index males |
|  |  | Body mass index |
|  |  | Age at menarche |
|  |  | Arm fat mass left |
|  |  | Arm fat mass right |
|  |  | Arm fat percentage left |
|  |  | Arm fat percentage right |
|  |  | Arm fat-free mass left |
|  |  | Arm fat-free mass right |
|  |  | Arm predicted mass left |
|  |  | Arm predicted mass right |
|  |  | Basal metabolic rate |
|  |  | Body fat percentage |
|  |  | Body mass index |
|  |  | Comparative body size at age 10 |
|  |  | Comparative height size at age 10 |
|  |  | Hip circumference |
|  |  | Impedance of arm left |
|  |  | Impedance of arm right |
|  |  | Impedance of leg left |
|  |  | Impedance of leg right |
|  |  | Impedance of whole body |
|  |  | Leg fat mass left |
|  |  | Leg fat mass right |
|  |  | Leg fat percentage left |
|  |  | Leg fat percentage right |
|  |  | Leg fat-free mass left |
|  |  | Leg fat-free mass right |
|  |  | Leg predicted mass left |
|  |  | Leg predicted mass right |
|  |  | Trunk fat mass |
|  |  | Trunk fat percentage |
|  |  | Trunk fat-free mass |
|  |  | Trunk predicted mass |
|  |  | Waist circumference |
|  |  | Weight |
|  |  | Whole body fat mass |
|  |  | Whole body fat-free mass |
|  |  | Whole body water mass |

| outcome | snp | confunder |
| --- | --- | --- |
| Tic disorder | rs11210887 | Educational attainment |
|  |  | Age at menarche |
|  |  | Age completed full time education |
|  |  | Current tobacco smoking |
|  |  | Fluid intelligence score |
|  |  | Qualifications: A levels or as levels or equivalent |
|  |  | Qualifications: college or university degree |
|  |  | Qualifications: none |
|  |  | Smoking status: current |
|  |  | Schizophrenia |
|  |  | Years of educational attainment |
|  | rs12705966 | Sleep duration |
|  |  | Types of physical activity in last 4 weeks: light diy |
|  |  | Wheeze or whistling in the chest in last year |
|  | rs4261436 | Intelligence multi trait analysis |
|  |  | Arm fat mass left |
|  |  | Arm fat mass right |
|  |  | Arm fat percentage left |
|  |  | Arm fat percentage right |
|  |  | Body fat percentage |
|  |  | Body mass index |
|  |  | Fluid intelligence score |
|  |  | Leg fat mass left |
|  |  | Leg fat mass right |
|  |  | Trunk fat mass |
|  |  | Whole body fat mass |
|  | rs4813421 | Arm fat-free mass left |
|  |  | Arm fat-free mass right |
|  |  | Arm predicted mass left |
|  |  | Arm predicted mass right |
|  |  | Basal metabolic rate |
|  |  | Trunk fat-free mass |
|  |  | Trunk predicted mass |
|  |  | Whole body fat-free mass |
|  |  | Whole body water mass |
|  | rs4916723 | Alcohol intake frequency |
|  | rs57806515 | Arm fat-free mass left |
|  |  | Arm predicted mass left |
|  |  | Basal metabolic rate |
|  |  | Comparative body size at age 10 |
|  |  | Impedance of arm left |
|  |  | Impedance of arm right |
|  |  | Impedance of leg left |
|  |  | Impedance of leg right |
|  |  | Impedance of whole body |
|  |  | Trunk fat-free mass |
|  |  | Trunk predicted mass |
|  |  | Whole body fat-free mass |
|  |  | Whole body water mass |
|  | rs704061 | Arm fat mass left |
|  |  | Arm fat mass right |
|  |  | Arm fat percentage left |
|  |  | Arm fat percentage right |
|  |  | Body fat percentage |
|  |  | Body mass index |
|  |  | Hip circumference |
|  |  | Leg fat mass left |
|  |  | Leg fat mass right |
|  |  | Leg fat percentage left |
|  |  | Leg fat percentage right |
|  |  | Leg fat-free mass left |
|  |  | Leg fat-free mass right |
|  |  | Leg predicted mass left |
|  |  | Number of operations |
|  |  | Overall health rating |
|  |  | Qualifications: college or university degree |
|  |  | Trunk fat mass |
|  |  | Trunk fat percentage |
|  |  | Waist circumference |
|  |  | Weight |
|  |  | Whole body fat mass |
|  | rs7563362 | Body mass index males |
|  |  | Body mass index |
|  |  | Age at menarche |
|  |  | Arm fat mass left |
|  |  | Arm fat mass right |
|  |  | Arm fat percentage left |
|  |  | Arm fat percentage right |
|  |  | Arm fat-free mass left |
|  |  | Arm fat-free mass right |
|  |  | Arm predicted mass left |
|  |  | Arm predicted mass right |
|  |  | Basal metabolic rate |
|  |  | Body fat percentage |
|  |  | Body mass index |
|  |  | Comparative body size at age 10 |
|  |  | Comparative height size at age 10 |
|  |  | Hip circumference |
|  |  | Impedance of arm left |
|  |  | Impedance of arm right |
|  |  | Impedance of leg left |
|  |  | Impedance of leg right |
|  |  | Impedance of whole body |
|  |  | Leg fat mass left |
|  |  | Leg fat mass right |
|  |  | Leg fat percentage left |
|  |  | Leg fat percentage right |
|  |  | Leg fat-free mass left |
|  |  | Leg fat-free mass right |
|  |  | Leg predicted mass left |
|  |  | Leg predicted mass right |
|  |  | Trunk fat mass |
|  |  | Trunk fat percentage |
|  |  | Trunk fat-free mass |
|  |  | Trunk predicted mass |
|  |  | Waist circumference |
|  |  | Weight |
|  |  | Whole body fat mass |
|  |  | Whole body fat-free mass |
|  |  | Whole body water mass |

| outcome | snp | trait |
| --- | --- | --- |
| Schizophrenia | rs11210887 | Educational attainment |
|  |  | Age at menarche |
|  |  | Age completed full time education |
|  |  | Current tobacco smoking |
|  |  | Fluid intelligence score |
|  |  | Qualifications: A levels or as levels or equivalent |
|  |  | Qualifications: college or university degree |
|  |  | Qualifications: none |
|  |  | Smoking status: current |
|  |  | Schizophrenia |
|  |  | Years of educational attainment |
|  | rs12705966 | Sleep duration |
|  |  | Types of physical activity in last 4 weeks: light diy |
|  |  | Wheeze or whistling in the chest in last year |
|  | rs4261436 | Intelligence multi trait analysis |
|  |  | Arm fat mass left |
|  |  | Arm fat mass right |
|  |  | Arm fat percentage left |
|  |  | Arm fat percentage right |
|  |  | Body fat percentage |
|  |  | Body mass index |
|  |  | Fluid intelligence score |
|  |  | Leg fat mass left |
|  |  | Leg fat mass right |
|  |  | Trunk fat mass |
|  |  | Whole body fat mass |
|  |  | Arm fat-free mass left |
|  |  | Arm fat-free mass right |
|  |  | Arm predicted mass left |
|  |  | Arm predicted mass right |
|  |  | Basal metabolic rate |
|  |  | Trunk fat-free mass |
|  |  | Trunk predicted mass |
|  |  | Whole body fat-free mass |
|  |  | Whole body water mass |
|  | rs704061 | Arm fat mass left |
|  |  | Arm fat mass right |
|  |  | Arm fat percentage left |
|  |  | Arm fat percentage right |
|  |  | Body fat percentage |
|  |  | Body mass index |
|  |  | Hip circumference |
|  |  | Leg fat mass left |
|  |  | Leg fat mass right |
|  |  | Leg fat percentage left |
|  |  | Leg fat percentage right |
|  |  | Leg fat-free mass left |
|  |  | Leg fat-free mass right |
|  |  | Leg predicted mass left |
|  |  | Number of operations |
|  |  | Overall health rating |
|  |  | Qualifications: college or university degree |
|  |  | Trunk fat mass |
|  |  | Trunk fat percentage |
|  |  | Waist circumference |
|  |  | Weight |
|  |  | Whole body fat mass |
|  | rs7563362 | Body mass index males |
|  |  | Body mass index |
|  |  | Age at menarche |
|  |  | Arm fat mass left |
|  |  | Arm fat mass right |
|  |  | Arm fat percentage left |
|  |  | Arm fat percentage right |
|  |  | Arm fat-free mass left |
|  |  | Arm fat-free mass right |
|  |  | Arm predicted mass left |
|  |  | Arm predicted mass right |
|  |  | Basal metabolic rate |
|  |  | Body fat percentage |
|  |  | Body mass index |
|  |  | Comparative body size at age 10 |
|  |  | Comparative height size at age 10 |
|  |  | Hip circumference |
|  |  | Impedance of arm left |
|  |  | Impedance of arm right |
|  |  | Impedance of leg left |
|  |  | Impedance of leg right |
|  |  | Impedance of whole body |
|  |  | Leg fat mass left |
|  |  | Leg fat mass right |
|  |  | Leg fat percentage left |
|  |  | Leg fat percentage right |
|  |  | Leg fat-free mass left |
|  |  | Leg fat-free mass right |
|  |  | Leg predicted mass left |
|  |  | Leg predicted mass right |
|  |  | Trunk fat mass |
|  |  | Trunk fat percentage |
|  |  | Trunk fat-free mass |
|  |  | Trunk predicted mass |
|  |  | Waist circumference |
|  |  | Weight |
|  |  | Whole body fat mass |
|  |  | Whole body fat-free mass |
|  |  | Whole body water mass |

| outcome | snp | trait |
| --- | --- | --- |
| Mood Disorders | rs11210887 | Educational attainment |
|  |  | Age at menarche |
|  |  | Age completed full time education |
|  |  | Current tobacco smoking |
|  |  | Fluid intelligence score |
|  |  | Qualifications: A levels or as levels or equivalent |
|  |  | Qualifications: college or university degree |
|  |  | Qualifications: none |
|  |  | Smoking status: current |
|  |  | Schizophrenia |
|  |  | Years of educational attainment |
|  | rs12705966 | Sleep duration |
|  |  | Types of physical activity in last 4 weeks: light diy |
|  |  | Wheeze or whistling in the chest in last year |
|  | rs4261436 | Intelligence multi trait analysis |
|  |  | Arm fat mass left |
|  |  | Arm fat mass right |
|  |  | Arm fat percentage left |
|  |  | Arm fat percentage right |
|  |  | Body fat percentage |
|  |  | Body mass index |
|  |  | Fluid intelligence score |
|  |  | Leg fat mass left |
|  |  | Leg fat mass right |
|  |  | Trunk fat mass |
|  |  | Whole body fat mass |
|  | rs4813421 | Arm fat-free mass left |
|  |  | Arm fat-free mass right |
|  |  | Arm predicted mass left |
|  |  | Arm predicted mass right |
|  |  | Basal metabolic rate |
|  |  | Trunk fat-free mass |
|  |  | Trunk predicted mass |
|  |  | Whole body fat-free mass |
|  |  | Whole body water mass |
|  | rs4916723 | Alcohol intake frequency |
|  | rs57806515 | Arm fat-free mass left |
|  |  | Arm predicted mass left |
|  |  | Basal metabolic rate |
|  |  | Comparative body size at age 10 |
|  |  | Impedance of arm left |
|  |  | Impedance of arm right |
|  |  | Impedance of leg left |
|  |  | Impedance of leg right |
|  |  | Impedance of whole body |
|  |  | Trunk fat-free mass |
|  |  | Trunk predicted mass |
|  |  | Whole body fat-free mass |
|  |  | Whole body water mass |
|  | rs704061 | Arm fat mass left |
|  |  | Arm fat mass right |
|  |  | Arm fat percentage left |
|  |  | Arm fat percentage right |
|  |  | Body fat percentage |
|  |  | Body mass index |
|  |  | Hip circumference |
|  |  | Leg fat mass left |
|  |  | Leg fat mass right |
|  |  | Leg fat percentage left |
|  |  | Leg fat percentage right |
|  |  | Leg fat-free mass left |
|  |  | Leg fat-free mass right |
|  |  | Leg predicted mass left |
|  |  | Number of operations |
|  |  | Overall health rating |
|  |  | Qualifications: college or university degree |
|  |  | Trunk fat mass |
|  |  | Trunk fat percentage |
|  |  | Waist circumference |
|  |  | Weight |
|  |  | Whole body fat mass |
|  | rs7563362 | Body mass index males |
|  |  | Body mass index |
|  |  | Age at menarche |
|  |  | Arm fat mass left |
|  |  | Arm fat mass right |
|  |  | Arm fat percentage left |
|  |  | Arm fat percentage right |
|  |  | Arm fat-free mass left |
|  |  | Arm fat-free mass right |
|  |  | Arm predicted mass left |
|  |  | Arm predicted mass right |
|  |  | Basal metabolic rate |
|  |  | Body fat percentage |
|  |  | Body mass index |
|  |  | Comparative body size at age 10 |
|  |  | Comparative height size at age 10 |
|  |  | Hip circumference |
|  |  | Impedance of arm left |
|  |  | Impedance of arm right |
|  |  | Impedance of leg left |
|  |  | Impedance of leg right |
|  |  | Impedance of whole body |
|  |  | Leg fat mass left |
|  |  | Leg fat mass right |
|  |  | Leg fat percentage left |
|  |  | Leg fat percentage right |
|  |  | Leg fat-free mass left |
|  |  | Leg fat-free mass right |
|  |  | Leg predicted mass left |
|  |  | Leg predicted mass right |
|  |  | Trunk fat mass |
|  |  | Trunk fat percentage |
|  |  | Trunk fat-free mass |
|  |  | Trunk predicted mass |
|  |  | Waist circumference |
|  |  | Weight |
|  |  | Whole body fat mass |
|  |  | Whole body fat-free mass |
|  |  | Whole body water mass |

| outcome | snp | trait |
| --- | --- | --- |
| Anxiety Disorder | rs11210887 | Educational attainment |
|  |  | Age at menarche |
|  |  | Age completed full time education |
|  |  | Current tobacco smoking |
|  |  | Fluid intelligence score |
|  |  | Qualifications: A levels or as levels or equivalent |
|  |  | Qualifications: college or university degree |
|  |  | Qualifications: none |
|  |  | Smoking status: current |
|  |  | Schizophrenia |
|  |  | Years of educational attainment |
|  | rs12705966 | Sleep duration |
|  |  | Types of physical activity in last 4 weeks: light diy |
|  |  | Wheeze or whistling in the chest in last year |
|  | rs4261436 | Intelligence multi trait analysis |
|  |  | Arm fat mass left |
|  |  | Arm fat mass right |
|  |  | Arm fat percentage left |
|  |  | Arm fat percentage right |
|  |  | Body fat percentage |
|  |  | Body mass index |
|  |  | Fluid intelligence score |
|  |  | Leg fat mass left |
|  |  | Leg fat mass right |
|  |  | Trunk fat mass |
|  |  | Whole body fat mass |
|  | rs4813421 | Arm fat-free mass left |
|  |  | Arm fat-free mass right |
|  |  | Arm predicted mass left |
|  |  | Arm predicted mass right |
|  |  | Basal metabolic rate |
|  |  | Trunk fat-free mass |
|  |  | Trunk predicted mass |
|  |  | Whole body fat-free mass |
|  |  | Whole body water mass |
|  | rs4916723 | Alcohol intake frequency |
|  | rs57806515 | Arm fat-free mass left |
|  |  | Arm predicted mass left |
|  |  | Basal metabolic rate |
|  |  | Comparative body size at age 10 |
|  |  | Impedance of arm left |
|  |  | Impedance of arm right |
|  |  | Impedance of leg left |
|  |  | Impedance of leg right |
|  |  | Impedance of whole body |
|  |  | Trunk fat-free mass |
|  |  | Trunk predicted mass |
|  |  | Whole body fat-free mass |
|  |  | Whole body water mass |
|  | rs704061 | Arm fat mass left |
|  |  | Arm fat mass right |
|  |  | Arm fat percentage left |
|  |  | Arm fat percentage right |
|  |  | Body fat percentage |
|  |  | Body mass index |
|  |  | Hip circumference |
|  |  | Leg fat mass left |
|  |  | Leg fat mass right |
|  |  | Leg fat percentage left |
|  |  | Leg fat percentage right |
|  |  | Leg fat-free mass left |
|  |  | Leg fat-free mass right |
|  |  | Leg predicted mass left |
|  |  | Number of operations |
|  |  | Overall health rating |
|  |  | Qualifications: college or university degree |
|  |  | Trunk fat mass |
|  |  | Trunk fat percentage |
|  |  | Waist circumference |
|  |  | Weight |
|  |  | Whole body fat mass |
|  | rs7563362 | Body mass index males |
|  |  | Body mass index |
|  |  | Age at menarche |
|  |  | Arm fat mass left |
|  |  | Arm fat mass right |
|  |  | Arm fat percentage left |
|  |  | Arm fat percentage right |
|  |  | Arm fat-free mass left |
|  |  | Arm fat-free mass right |
|  |  | Arm predicted mass left |
|  |  | Arm predicted mass right |
|  |  | Basal metabolic rate |
|  |  | Body fat percentage |
|  |  | Body mass index |
|  |  | Comparative body size at age 10 |
|  |  | Comparative height size at age 10 |
|  |  | Hip circumference |
|  |  | Impedance of arm left |
|  |  | Impedance of arm right |
|  |  | Impedance of leg left |
|  |  | Impedance of leg right |
|  |  | Impedance of whole body |
|  |  | Leg fat mass left |
|  |  | Leg fat mass right |
|  |  | Leg fat percentage left |
|  |  | Leg fat percentage right |
|  |  | Leg fat-free mass left |
|  |  | Leg fat-free mass right |
|  |  | Leg predicted mass left |
|  |  | Leg predicted mass right |
|  |  | Trunk fat mass |
|  |  | Trunk fat percentage |
|  |  | Trunk fat-free mass |
|  |  | Trunk predicted mass |
|  |  | Waist circumference |
|  |  | Weight |
|  |  | Whole body fat mass |
|  |  | Whole body fat-free mass |
|  |  | Whole body water mass |
